# Supplementary figures and images for: Delaying surgery beyond six weeks after systemic therapy reduces postoperative morbidity without evidence of impaired oncologic outcomes in colorectal liver metastases
Source: BMC Cancer. 2026 Jun 30;26:786. doi: 10.1186/s12885-026-16386-4 (PMC13317304; doi:10.1186/s12885-026-16386-4)

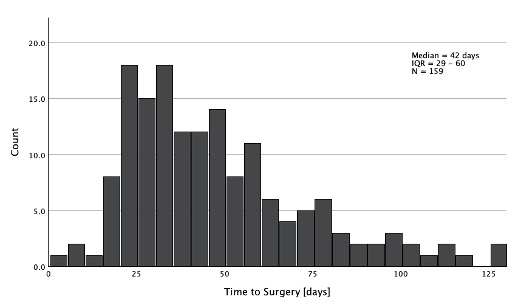

Supplement: Supplementary file 1 — Supplementary Material 1. [file 12885_2026_16386_MOESM1_ESM.jpg]

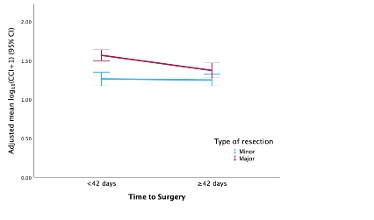

Supplement: Supplementary file 2 — Supplementary Material 2. [file 12885_2026_16386_MOESM2_ESM.jpg]
