# Supplementary material for: Delaying surgery beyond six weeks after systemic therapy reduces postoperative morbidity without evidence of impaired oncologic outcomes in colorectal liver metastases
Source: BMC Cancer. 2026 Jun 30;26:786. doi: 10.1186/s12885-026-16386-4 (PMC13317304; doi:10.1186/s12885-026-16386-4)
Supplement: Supplementary file 3 — Supplementary Material 3. [file 12885_2026_16386_MOESM3_ESM.docx]

**Original Article**

***Title.*** Delaying surgery beyond six weeks after systemic therapy reduces postoperative morbidity without evidence of impaired oncologic outcomes in colorectal liver metastases

***Author names and affiliations.*** Esther Giehl-Brown^1^, Rajan Nikbakhsh^1^, Ana Mansourkiaei^1^, Laila Jötten^1^, Bruno Christian Köhler^2^, Thomas Longerich^3^, Bo Kong^1^, Arianeb Mehrabi^1^, Markus W Büchler^4^, Mohammed Al-Saeedi^1#^, Christoph Kahlert^1#^

^1^Department of General, Visceral and Transplantation Surgery, University of Heidelberg, Heidelberg, Germany

^2^Liver Cancer Center Heidelberg, Heidelberg, Germany; Department of Medical Oncology, National Center for Tumor Diseases, Heidelberg University Hospital, Heidelberg, Germany.

^3^Institute of Pathology, University Hospital Heidelberg, Heidelberg 69120, Germany.

^4^Botton-Champalimaud Pancreatic Cancer Center, Champalimaud Foundation, Lisbon, Portugal.

^#^contributed equally to this work.

***Corresponding author and present address.***

Prof. Dr. med. Christoph Kahlert, MHBA, FEBS (Surgical Oncology)

Department of General, Visceral and Transplantation Surgery, University of Heidelberg

Im Neuenheimer Feld 420, 69120 Heidelberg, Germany

E-Mail: [Christoph.Kahlert@med.uni-heidelberg.de](mailto:Christoph.Kahlert@med.uni-heidelberg.de); Phone number: +49 6221 56-310222

***Disclosures.***

- ***Conflict of interest.*** The authors declare no conflicts of interest related to this manuscript.
- ***Funding****.* The authors have no funding to disclose for this study.
- ***Data availability.*** The data supporting the findings of this study are available upon reasonable request from the corresponding author.
- ***Application of AI.*** During the preparation of this work the authors used ChatGPT (OpenAI, San Francisco, CA, USA) in order to improve readability. After using this tool, the authors edited the content as needed and take full responsibility.

**Abstract**

Background: The optimal timing of resection for colorectal liver metastases (CRLM) after systemic therapy remains unclear. This study evaluated the impact of time-to-surgery (TTS) on postoperative morbidity and oncologic outcomes.

Methods: In this retrospective cohort (2018–2022) from a German high‑volume hepatobiliary center, 159 patients underwent hepatic resection for CRLM following systemic therapy. Patients were stratified by TTS ≤41 versus ≥42 days. The primary endpoint was clinically meaningful postoperative morbidity (Comprehensive Complication Index ≥ 30). Secondary endpoints included liver-specific recurrence-free survival (RFS) and overall survival (OS). Multivariable regression, Cox proportional-hazards models, and prespecified subgroup analyses were performed.

Results: CCI ≥30 occurred in 48.7% of patients with shorter TTS versus 31.3% with longer TTS (P = 0.023). After multivariable adjustment, TTS ≥42 days was independently associated with lower odds of postoperative morbidity (OR 0.355; 95% CI 0.127–0.992; P = 0.048). In the subgroup of major hepatectomies, TTS ≥42 days demonstrated an even more pronounced protective effect (OR 0.069; 95% CI 0.006–0.778; P = 0.031). However, TTS ≥42 days was not independently associated with liver-specific RFS or OS.

Conclusion: Delaying surgery to ≥6 weeks after neoadjuvant systemic therapy was associated with significantly reduced postoperative morbidity without evidence of impaired early oncologic outcomes among patients who ultimately underwent resection. These findings support consideration of a prolonged interval before complex hepatectomy while highlighting the need for prospective validation.

Keywords: Colorectal liver metastases – Oncologic liver surgery – Neoadjuvant systemic therapy – Postoperative morbidity – Time to surgery interval.

**Introduction**

The liver is the most common site of distant metastasis in colorectal cancer, with synchronous colorectal liver metastases (CRLM) present in up to 20% at diagnosis.(1) In combination with neoadjuvant systemic therapy (NAT), surgical resection remains the only potentially curative treatment option for patients with CRLM.(2, 3) Advances in liver surgery, including parenchymal-sparing techniques, and two-stage hepatectomy, have further increased the number of patients eligible for potentially curative treatment.(4) Despite these advances, long-term disease control remains limited, with a large single-institution series reporting a 10-year recurrence-free survival (RFS) or a minimum of three years of disease-free interval in only 20% of patients following resection.(5)

Systemic therapy is the standard of care for patients with initially unresectable CRLM, potentially downstaging disease to enable surgical resection. However, the role of NAT in patients with initially resectable CRLM remains controversial due to the lack of consistent evidence for a survival benefit. Potential advantages of NAT include eradication of micrometastases, improved margin-negative resection rates, tumor shrinkage, and in vivo assessment of chemosensitivity.(6) Furthermore, first-line chemotherapy combined with targeted agents have demonstrated benefits in RFS when applied in the perioperative setting.(7)

Optimal timing of surgery after NAT (time-to-surgery; TTS) has yet to be defined, but may critically influence both postoperative morbidity and long-term outcomes. NAT-associated hepatotoxicity, including sinusoidal obstruction and steatohepatitis, has been linked to increased surgical morbidity and may support a brief delay to permit hepatic recovery.(8, 9) Conversely, prolonged intervals may allow residual tumor cells to proliferate, potentially increasing recurrence risk.(10) Few studies have directly addressed optimal TTS. A retrospective cohort study from China suggested that surgery at 4–6 weeks following NAT improved survival versus 6–8 weeks, despite higher complication rates.(10) Importantly, postoperative morbidity itself has been identified as an independent predictor of reduced survival in CRLM.(11-13).

We therefore conducted a retrospective analysis of 159 patients at a high-volume tertiary center to evaluate the impact of TTS on postoperative morbidity and oncologic outcomes following surgery for CRLM. By clarifying the impact of surgical timing, this study aims to inform perioperative decision-making and optimise the balance between operative safety and oncologic efficacy.

**Methods**

- *Study design and setting*

This retrospective cohort study included adult patients (≥ 18 years) who underwent oncologic resection of CRLM following NAT at Heidelberg University Hospital, a high‑volume hepatobiliary center in Germany. A total of 159 consecutive patients treated between January 1, 2018 and December 31, 2022 were identified through the National Center for Tumor Diseases (NCT) Cancer Registry Heidelberg. The study was conducted in accordance with the Declaration of Helsinki and approved by the Ethics Committee of the Medical Faculty, University of Heidelberg, Germany (reference number S-066-2024). Patient consent was waived due to the retrospective and anonymized nature of the study.

- *Participants*

Eligible patients had histologically confirmed synchronous or metachronous CRLM and received NAT prior to hepatic resection. From an initial 324 cases identified, 165 were excluded owing to absence of documented systemic therapy (n = 151), non‑resection (n = 8), or duplicate records (n = 6), leaving 159 patients for analysis (CONSORT flow diagram). Last surveillance was performed in August 2024.

*Variables*

The primary exposure variable was TTS, defined as the number of days between the final NAT dose and the date of surgery. TTS was analyzed both continuously and dichotomized at ≥ 35, 42, 49, 56 days. This cut-off was selected based on biological and clinical considerations and aligned with the median TTS. Previous investigations have demonstrated clinically relevant differences in postoperative outcomes within this interval range, including Wang et al., who observed differences in morbidity and survival between 4–6 and 6–8 weeks after NAT,(10) and Nordlinger et al., who incorporated a 6-week interval into perioperative systemic therapy protocols in resectable CRLM.(7) Sensitivity analyses using alternative dichotomization thresholds (35, 49, and 56 days) were performed to assess the robustness of the findings.

To minimize arbitrary categorization, TTS was additionally modeled as a continuous variable using restricted cubic splines.

The primary outcome was clinically meaningful postoperative morbidity, operationalized as a Comprehensive Complication Index (CCI) > 30 within 90 days of surgery.(14, 15) Secondary outcomes were liver‑specific RFS and OS. Recurrence was defined radiologically or histologically.

- *Data Sources and Bias*

Patient demographics, perioperative details, and outcomes were abstracted by two independent reviewers from the NCT registry and institutional database. To minimize information bias, all complications were graded independently by two clinicians unaware of TTS. Major hepatectomy was defined as the resection of ≥3 Couinaud segments, including two-stage or ALPPS hepatectomy. Molecular data (KRAS, BRAF mutation status) was obtained from pathology reports. Variables with missing data in fewer than 5% of cases were retained in the analyses. Potential confounding was addressed by prespecifying adjustment for clinically relevant covariates in multivariable models. Inverse‑probability‑of‑treatment weighting (IPTW) was applied in sensitivity analyses to further balance baseline characteristics across TTS groups.

- *Statistical Methods*

Continuous variables are presented as medians with IQR and compared using the Mann–Whitney U test. Categorical variables are reported as counts and percentages and compared using χ² or Fisher’s exact test. Univariable and multivariable logistic regression models estimated odds ratios (ORs) and 95% confidence intervals (CIs) for CCI > 30, incorporating both dichotomized and continuous TTS. The continuous analysis employed restricted cubic splines with knots at the 10th, 50th, and 90th percentiles (12, 42, and 89 days) to assess non‑linear effects. Prespecified covariates were retained in multivariable models irrespective of univariable significance, and subgroup analyses focused on patients undergoing major hepatectomy. Survival outcomes were evaluated by Kaplan–Meier curves with log‑rank tests. Cox proportional‑hazards models provided hazard ratios (HRs) with 95% CIs. All tests were two‑sided with P < 0.05 denoting significance. Analyses were performed using SPSS Statistics version 23 (IBM Corp., Armonk, NY, USA) and R version 4.4.2 (R Foundation for Statistical Computing, Vienna, Austria).

**Results**

- *Cohort characteristics*

A total of 159 patients underwent hepatic resection for CRLM after systemic therapy. The median TTS was 42 days (IQR 29–63; Table 1, Supplementary Figure 1). FOLFOX regimen was more frequently administered in patients with TTS ≥ 42 days (44.6% vs. 27.6%; P = 0.032), whereas FOLFOXIRI was more commonly used with TTS < 42 days (25.0% vs. 12.0%; P = 0.041; Table 1). The median number of cycles was six (IQR 5–8). Treatment intent was neoadjuvant in 86.2% and conversion in 10.7% (P = 0.127), with 15.7% discontinuing early (3.8% for progression, 11.9% for toxicity). Median age was 59 years (IQR 51–67), 64.8% were male. ASA grades I–II comprised 49.7% versus III–IV in 46.5%. Synchronous metastases were present in 83.0%, bilobar disease in 72.3%. KRAS mutations were observed in 42.8%, and an R0 margin was achieved in 83.0%. Most resections were performed via an open approach (80.5%), minimally invasive only in 16.4%. Atypical resections accounted for 45.9%. Simultaneous extrahepatic resections were performed in 38.4% (Table 1).

- *Postoperative outcomes*

The overall median postoperative hospital stay was 13.5 days (IQR 9–23), with no significant difference between the shorter‑ and longer‑TTS groups (15 versus 13 days; P = 0.242, Table 2). Similarly, median intensive care unit stay did not differ significantly (1 versus 2 days; P = 0.073). The incidence of surgical‑site infections was 31.6% in the short‑TTS group compared with 20.5% in the long‑TTS group (P = 0.105), while rates of postoperative hemorrhage (10.5% versus 8.4%; P = 0.609), biliary leakage (30.3% versus 26.5%; P = 0.598), and post‑hepatectomy liver failure (5.3% versus 2.4%; P = 0.421) were similar. Reoperation rates were comparable (15.8% versus 16.9%; P = 1.000). Thirty‑day readmission occurred in 22.4% of patients with TTS < 42 days versus 10.8% with TTS ≥ 42 days (P = 0.051), and the pattern persisted at 90 days (19.7% versus 10.8%; P = 0.120). Ninety‑day mortality remained low at 0.6% overall and showed no difference by TTS (P = 0.478, Table 2).

- *Predictive Factors for Clinically Meaningful Morbidity*

Clinically significant postoperative morbidity (CCI ≥ 30) occurred in 48.7% of patients with TTS < 42 days versus 31.3% of those with TTS ≥ 42 days (P = 0.023; Table 2).

In univariable logistic regression, a delay of ≥42 days was associated with reduced odds of clinically significant morbidity (CCI ≥30) (OR 0.464; 95% CI 0.242–0.890; P = 0.021; Table 3A). Major hepatectomy (OR 3.412; 95% CI 1.748–6.661; P < 0.001), bilobar disease (OR 3.054; 95% CI 1.377–6.775; P = 0.006), synchronous metastases (OR 3.594; 95% CI 1.282–10.079; P = 0.015), and open approach compared with minimally invasive surgery (OR 0.254; 95% CI 0.082–0.789; P = 0.018, Table 3A) were significantly associated with morbidity. After multivariable adjustment, TTS ≥ 42 days remained independently associated with lower odds of clinically significant morbidity (OR 0.355; 95% CI 0.127–0.992; P = 0.048, Table 3A). Major hepatectomy (OR 5.487; 95% CI 1.846–16.307; P = 0.002), bilobar distribution (OR 4.576; 95% CI 1.155–18.132; P = 0.030), and the number of systemic therapy cycles (OR 1.189; 95% CI 1.030–1.373; P = 0.018) also remained statistically significant, whereas bevacizumab use was not independently associated with CCI ≥30 (OR 0.451; 95% CI 0.148–1.373; P = 0.161, Table 3A).

When TTS was modeled as a continuous variable, each additional day was associated with reduced odds of clinically significant morbidity in univariable analysis (OR 0.986 per day; 95% CI 0.973–1.000; P = 0.045; Table 3B). However, this association was no longer statistically significant after multivariable adjustment (OR 0.985 per day; 95% CI 0.965–1.005; P = 0.136; Table 3B). In this model, major hepatectomy (OR 5.125; 95% CI 1.776–14.788; P = 0.003), bilobar disease (OR 4.606; 95% CI 1.186–17.898; P = 0.027), and number of systemic therapy cycles (OR 1.173 per cycle; 95% CI 1.017–1.354; P = 0.029; Table 3B) remained independently associated with postoperative morbidity.

Restricted cubic spline analysis with knots at days 12, 42, and 89 demonstrated no evidence of nonlinearity (P = 0.543 and 0.429 for spline terms; Supplementary Table 1).

Sensitivity analyses using alternative TTS cut-offs confirmed a consistent inverse association between longer TTS and clinically significant postoperative morbidity after multivariable adjustment, with statistical significance observed for TTS ≥49 days (OR 0.346, 95% CI 0.122–0.985; P = 0.047), whereas TTS ≥35 days (P = 0.166) and TTS ≥56 days (P = 0.061) were not statistically significant (Supplementary Table 4).

In the major hepatectomy subgroup, TTS ≥42 days was independently associated with lower odds of clinically significant morbidity after adjustment (0.069; 95% CI 0.006–0.778; P = 0.031; Table 3C). When modeled as a continuous variable, TTS showed a similar but non-significant trend (adjusted OR 0.953 per day; 95% CI 0.908–1.001; P = 0.057; Table 3D). In the minor hepatectomy subgroup, clinically significant morbidity (CCI >30) occurred in n=24 (27.6%) of patients; no independent predictors were identified (data not shown).

To address potential selection bias, inverse probability of treatment weighting (IPTW)–weighted linear regression of log₁₀(CCI + 1) demonstrated a significant interaction between TTS and resection extent (Supplementary Table 2B). Major resections performed <42 days after NAT were associated with higher postoperative morbidity compared with those performed ≥42 days (B = 0.304; 95% CI 0.187–0.421; P <0.001; Supplementary Table 2B), whereas no difference was observed for minor resections (B = −0.014; 95% CI −0.123 to 0.095; P = 0.798). Adjusted marginal means corresponded to an approximate 11-point absolute difference in CCI for major resections (24 vs. 35), with minimal difference for minor resections (18 vs. 19) (Supplementary Table 2A, C; Supplementary Figure 2).

- *Predictive Factors for Liver-specific RFS and OS*

Over a median follow-up of 31.6 months, 96 of 159 patients (60.4 %) experienced liver-specific recurrence. Median liver-specific RFS was 8.4 months for TTS < 42 days versus 9.9 months for TTS ≥ 42 days (log-rank P = 0.275; Figure 1). In univariable Cox regression using dichotomized TTS, a delay of ≥ 42 days showed a nonsignificant trend for liver-specific RFS (HR 0.688; 95 % CI 0.460–1.030; P = 0.069; Table 4A). After multivariable adjustment, only two-stage hepatectomy remained independently associated with earlier relapse (HR 2.228; 95 % CI 1.146–4.329; P = 0.018; Table 4A). When TTS was modeled as a continuous variable, no association with liver-specific RFS was observed in either univariable (HR 1.006 per day; 95 % CI 0.998–1.015; P = 0.156) or multivariable analyses (HR 0.999; 95 % CI 0.985–1.013; P = 0.867; Table 4B). Two-stage hepatectomy remained the only independent predictor of shorter liver-specific RFS (HR 2.282; 95 % CI 1.188–4.386; P = 0.013, Table 4B).

Kaplan–Meier analysis over the follow-up period confirmed no significant difference in liver-specific RFS between TTS groups (log-rank χ² = 3.35; P = 0.067; Figure 1). Median OS did not differ according to TTS subgroup (26.4 vs. 31.3 months; log-rank P = 0.530) and remained non-significant after multivariable adjustment (Supplementary Table 3).

**Discussion**

Surgical timing within the multidisciplinary treatment of CRLM is clinically relevant to maximize synergistic therapeutic effects while limiting adverse interactions between systemic therapy and hepatic resection. Evidence on the impact of TTS in CRLM is limited. In this retrospective cohort study of predominantly initially resectable CRLM, we evaluated the association between TTS and postoperative outcomes using TTS as both a continuous variable and across multiple dichotomized cut-offs, including ≥42 days.

The selection of a 42-day cut-off warrants specific consideration. This interval is biologically and clinically meaningful. Systemic therapy-associated liver injury peaks shortly after treatment cessation and partially recovers within 6–8 weeks. From a clinical perspective, perioperative systemic therapy trials such as the EORTC 40983 study by Nordlinger et al. incorporated treatment-free intervals of approximately six weeks before surgery, establishing this timeframe as a pragmatic standard in multidisciplinary management of CRLM.(7) Wang et al. demonstrated differences in postoperative morbidity and survival between 4–6 and 6–8 weeks,(10) whereas Chen et al. examined substantially longer delays (>3 months), reflecting a different clinical context.(16)

We found that a shorter TTS was associated with significantly higher rates of clinically meaningful postoperative morbidity particularly in patients undergoing major resections. Although spline-adjusted analyses did not demonstrate a gradual or non-linear association across the full TTS range, dichotomization at 42 days revealed a clinically relevant threshold effect. Patients with TTS ≥42 days had significantly lower odds of CCI ≥30. From a pathophysiological perspective, systemic therapy‑associated liver injury, steatosis and steatohepatitis from irinotecan, and sinusoidal obstruction syndrome from oxaliplatin, may partially explain this finding, given their gradual resolution over several weeks.(8, 9, 17) Histologic evidence suggests a 6–8 weeks interval allows resolution of sinusoidal and hepatocellular injury. Delaying resection beyond 42 days likely permits hepatic regeneration, reducing bleeding risk and infection. In addition, anti-VEGF therapy such as bevacizumab has been associated with impaired wound healing and vascular repair,(18) providing further biological rationale for allowing an adequate recovery interval before major hepatectomy. However, in our extended multivariable analyses including bevacizumab exposure, longer TTS remained independently associated with reduced postoperative morbidity, and bevacizumab itself was not an independent predictor of clinically significant complications. Patients with shorter TTS had higher surgical‑site infection rates, greater median CCI, and a trend toward increased 30‑day readmissions.

The absence of a significant association between TTS and postoperative morbidity in minor hepatectomy may reflect true clinical equivalence but may also be influenced by lower event rates and reduced statistical power. In contrast, major hepatectomy represents a higher-risk context in which recovery from systemic therapy and restoration of hepatic functional reserve are likely more critical determinants of outcome.

Beyond fixed chronological thresholds, individualized assessment of hepatic functional reserve may further refine surgical timing after NAT. Functional liver tests such as 99mTc-based hepatobiliary scintigraphy allow regional quantification of liver function and regenerative capacity. Recent multicentre data by Xiao et al. demonstrated that functional future liver remnant parameters derived from 99mTc-GSA SPECT/CT independently predicted post-hepatectomy liver failure in patients undergoing major liver resection.(19) These findings suggest that functional imaging could support a function-guided approach to surgical scheduling, particularly prior to major hepatectomy.

The benefit of NAT for initially resectable CRLM remains debated, as clear survival benefits have not been consistently demonstrated.(6) Several studies have suggested that earlier surgery following systemic therapy may be associated with favorable oncologic outcomes, despite higher postoperative complication rates. Importantly, delayed surgery did not adversely affect early oncologic outcomes in our cohort. Although hepatic recurrence was more frequent among patients with shorter TTS, median liver-specific RFS and OS were similar between groups. Wang et al. reported improved DFS and OS in patients undergoing resection within 4–6 weeks compared with 6–8 weeks,(10) while Sutton et al. demonstrated that shorter intervals between NAT and surgery were associated with improved oncologic outcomes in breast cancer.(20) Together, these findings highlight a potential trade-off between oncologic efficacy and perioperative safety. In contrast, our data indicate that extending the interval beyond six weeks substantially reduces postoperative morbidity, without evidence of impaired early liver-specific RFS or OS, acknowledging that the study may be underpowered to detect smaller but clinically meaningful differences.

In contrast to prior studies reporting overall RFS, we selected liver-specific RFS as a secondary oncologic endpoint. This decision was based on the premise that TTS is more likely to influence intrahepatic outcomes through chemotherapy-associated liver injury, hepatic regeneration, and perioperative morbidity, rather than systemic tumor dissemination. Extrahepatic recurrences are predominantly determined by tumor biology and response to systemic therapy and may therefore obscure associations, related to surgical timing.(12, 13) By focusing on liver-specific recurrence, we aimed to assess oncologic outcomes most directly linked to hepatic resection and perioperative recovery.

Perioperative blood transfusions, often reflecting surgical complexity and complications, are linked to impaired OS after liver resection.(21) Reducing perioperative morbidity through optimal TTS may thus improve both short‑ and long‑term outcomes. Conversely, an international multicenter study of nearly 5,000 minimally invasive minor hepatectomies for CRLM showed no increase in perioperative morbidity after NAT.(22) This discrepancy likely reflects differences in surgical risk, with minor resections tolerating shorter intervals, whereas major hepatectomies benefit from extended hepatic recovery.

While very prolonged delays to surgery have been associated with worse OS in other cohorts, as reported by Chen et al., we observed no association between TTS and OS within the clinically relevant interval examined in our study.(16) However, an important counterargument to prolonging the interval between NAT and hepatectomy is the risk of interval disease progression, which may render some patients unresectable. Because our cohort included only patients who ultimately underwent resection, our analysis is inherently subject to selection bias and cannot quantify progression to unresectability during the TTS interval period. Accordingly, the absence of inferior oncologic outcomes should not be interpreted as evidence that delaying surgery is oncological neutral.

This study has several strengths, including a contemporary, well‑characterized cohort treated under standardized protocols in a high‑volume tertiary center; detailed multivariable and inverse‑probability weighting analyses; and procedure‑specific subgroup evaluation. However, limitations include the retrospective, single‑center design, which limits causal inference and generalizability, as unmeasured factors such as patient preference and institutional scheduling could influence TTS. Heterogeneity in chemotherapy regimens and biologic agents adds further confounding, though we adjusted for key treatment variables. Finally, the cohort size may be underpowered to detect smaller survival differences.

Moreover, TTS is influenced by multiple clinical and logistical factors, including patient recovery, tumor aggressiveness, surgical complexity, and institutional scheduling. Despite multivariable adjustment and inverse probability weighting, residual confounding and indication bias cannot be fully excluded. In particular, the higher rate of liver-specific recurrence in the shorter-TTS group raises the possibility that patients with more biologically aggressive disease were preferentially scheduled for earlier surgery, underscoring that these associations should not be interpreted as causal. In addition, the number of outcome events relative to the number of covariates may increase the risk of model overfitting, and the multivariable analyses should therefore be interpreted as exploratory despite supporting sensitivity analyses.

In conclusion, surgical timing appears to be a modifiable factor influencing perioperative safety in CRLM. Delaying major hepatic resection to ≥6 weeks after NAT was associated with lower odds of clinically significant morbidity, without evidence of impaired early liver-specific RFS or OS among patients who ultimately underwent resection. No comparable effect was observed for minor resections, suggesting that optimal timing strategies may differ according to surgical extent. These findings support a personalized approach to surgical scheduling that integrates tumor biology, surgical complexity, and hepatic functional recovery rather than a uniform delay strategy. Prospective, adequately powered multicenter studies incorporating contemporary systemic therapies, standardized timing protocols, and functional liver assessment are required to establish evidence-based guidelines for optimal surgical timing in CRLM.

**References**

1. Valderrama-Treviño AI, Barrera-Mera B, Ceballos-Villalva JC, Montalvo-Javé EE. Hepatic Metastasis from Colorectal Cancer. Euroasian J Hepatogastroenterol. 2017;7(2):166-75.

2. Osterlund P, Salminen T, Soveri LM, Kallio R, Kellokumpu I, Lamminmäki A, et al. Repeated centralized multidisciplinary team assessment of resectability, clinical behavior, and outcomes in 1086 Finnish metastatic colorectal cancer patients (RAXO): A nationwide prospective intervention study. Lancet Reg Health Eur. 2021;3:100049.

3. Tomlinson JS, Jarnagin WR, DeMatteo RP, Fong Y, Kornprat P, Gonen M, et al. Actual 10-year survival after resection of colorectal liver metastases defines cure. J Clin Oncol. 2007;25(29):4575-80.

4. Petrowsky H, Linecker M, Raptis DA, Kuemmerli C, Fritsch R, Kirimker OE, et al. First Long-term Oncologic Results of the ALPPS Procedure in a Large Cohort of Patients With Colorectal Liver Metastases. Ann Surg. 2020;272(5):793-800.

5. Creasy JM, Sadot E, Koerkamp BG, Chou JF, Gonen M, Kemeny NE, et al. Actual 10-year survival after hepatic resection of colorectal liver metastases: what factors preclude cure? Surgery. 2018;163(6):1238-44.

6. Nigri G, Petrucciani N, Ferla F, La Torre M, Aurello P, Ramacciato G. Neoadjuvant chemotherapy for resectable colorectal liver metastases: what is the evidence? Results of a systematic review of comparative studies. Surgeon. 2015;13(2):83-90.

7. Nordlinger B, Sorbye H, Glimelius B, Poston GJ, Schlag PM, Rougier P, et al. Perioperative chemotherapy with FOLFOX4 and surgery versus surgery alone for resectable liver metastases from colorectal cancer (EORTC Intergroup trial 40983): a randomised controlled trial. Lancet. 2008;371(9617):1007-16.

8. Rubbia-Brandt L, Audard V, Sartoretti P, Roth AD, Brezault C, Le Charpentier M, et al. Severe hepatic sinusoidal obstruction associated with oxaliplatin-based chemotherapy in patients with metastatic colorectal cancer. Ann Oncol. 2004;15(3):460-6.

9. Vauthey JN, Pawlik TM, Ribero D, Wu TT, Zorzi D, Hoff PM, et al. Chemotherapy regimen predicts steatohepatitis and an increase in 90-day mortality after surgery for hepatic colorectal metastases. J Clin Oncol. 2006;24(13):2065-72.

10. Wang Y, Wen N, Xiong X, Lu J, Li B, Cheng N. Timing of surgery in patients with synchronous colorectal cancer liver metastases undergoing neoadjuvant chemotherapy: a propensity score analysis. World J Surg Oncol. 2023;21(1):276.

11. Laurent C, Sa Cunha A, Couderc P, Rullier E, Saric J. Influence of postoperative morbidity on long-term survival following liver resection for colorectal metastases. Br J Surg. 2003;90(9):1131-6.

12. D'Angelica M, Kornprat P, Gonen M, DeMatteo RP, Fong Y, Blumgart LH, et al. Effect on outcome of recurrence patterns after hepatectomy for colorectal metastases. Ann Surg Oncol. 2011;18(4):1096-103.

13. Viganò L, Ferrero A, Lo Tesoriere R, Capussotti L. Liver surgery for colorectal metastases: results after 10 years of follow-up. Long-term survivors, late recurrences, and prognostic role of morbidity. Ann Surg Oncol. 2008;15(9):2458-64.

14. Dindo D, Demartines N, Clavien PA. Classification of surgical complications: a new proposal with evaluation in a cohort of 6336 patients and results of a survey. Ann Surg. 2004;240(2):205-13.

15. Slankamenac K, Nederlof N, Pessaux P, de Jonge J, Wijnhoven BP, Breitenstein S, et al. The comprehensive complication index: a novel and more sensitive endpoint for assessing outcome and reducing sample size in randomized controlled trials. Ann Surg. 2014;260(5):757-62; discussion 62-3.

16. Chen EY, Mayo SC, Sutton T, Kearney MR, Kardosh A, Vaccaro GM, et al. Effect of Time to Surgery of Colorectal Liver Metastases on Survival. J Gastrointest Cancer. 2021;52(1):169-76.

17. Ramadori G, Cameron S. Effects of systemic chemotherapy on the liver. Ann Hepatol. 2010;9(2):133-43.

18. Scappaticci FA, Fehrenbacher L, Cartwright T, Hainsworth JD, Heim W, Berlin J, et al. Surgical wound healing complications in metastatic colorectal cancer patients treated with bevacizumab. J Surg Oncol. 2005;91(3):173-80.

19. Xiao M, Song J, Lyu Z, Huang X, Zhang Y, Wang L, et al. Regional Liver Function Assessment Using. Ann Surg Oncol. 2025;32(9):6230-42.

20. Sutton TL, Schlitt A, Gardiner SK, Johnson N, Garreau JR. Time to surgery following neoadjuvant chemotherapy for breast cancer impacts residual cancer burden, recurrence, and survival. J Surg Oncol. 2020;122(8):1761-9.

21. Giehl-Brown E, Geipel E, Löck S, Dehlke K, Schweipert J, Weitz J, et al. Transfusions of packed red blood cells in surgery for liver cancer: predictor of impaired overall survival but not recurrence-free survival - impact of blood transfusions in liver surgery. J Gastrointest Surg. 2024;28(4):402-11.

22. Hoogteijling TJ, Abu Hilal M, Zimmitti G, Aghayan DL, Wu AGR, Cipriani F, et al. Impact of neoadjuvant chemotherapy on short-term outcomes after simple and complex minimally invasive minor hepatectomy for colorectal liver metastases: A propensity-score matched and coarsened exact matched study. Eur J Surg Oncol. 2024;50(6):108309.

**Figure 1.** Kaplan–Meier estimates of liver‑specific recurrence‑free survival (RFS) for patients undergoing hepatic resection following neoadjuvant chemotherapy, stratified by a shorter (≤ 41 days; blue line) versus longer (≥ 42 days; red line) time‑to‑surgery (TTS) interval. Over the entire follow‑up period (median 40.5 months), there was no statistically significant difference between the two groups (log‑rank χ² = 3.35; P = 0.067).


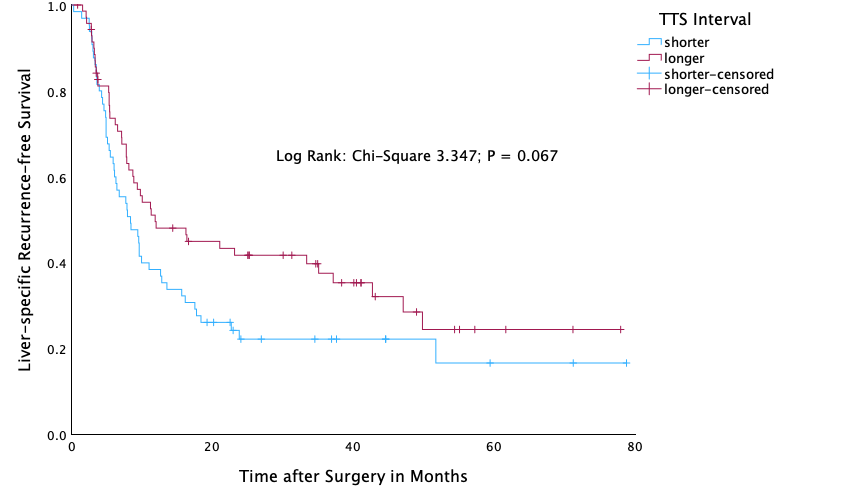


**Table 1.** Demographic and clinical characteristics of the study cohort (n = 159), stratified by time-to-surgery (TTS) ≤ 41 days (Shorter TTS, n = 76) versus ≥ 42 days (Longer TTS, n = 83).

Data are presented as median (interquartile range) for continuous variables and number (percentage) for categorical variables. P-values compare the two TTS groups using the Mann–Whitney U test for continuous variables and χ² or Fisher’s exact test for categorical variables, as appropriate.

Abbreviations: BMI, body mass index; ASA, American Society of Anesthesiologists; CEA, carcinoembryonic antigen; FOLFOX, 5-fluorouracil/leucovorin/oxaliplatin; FOLFIRI, 5-fluorouracil/leucovorin/irinotecan.

|  | **Total**  **[n=159]** | **Shorter TTS**  **[n=76]** | **Longer TTS**  **[n=83]** |  |
| --- | --- | --- | --- | --- |
| **Demographic data** | **Median (IQR) or Number (Percentage)** | | | **P** |
| Age | 59 (51-67) | 58 (51.3-65) | 59 (51-67) | 0.763 |
| Male gender | 103 (64.8%) | 51 (67.1%) | 52 (62.7%) | 0.619 |
| BMI | 25.6 (22.6-27.8) | 25.9 (23.2-28.8) | 25.0 (22.0-27.6) | 0.181 |
| ASA |  |  |  |  |
| I/II | 79 (49.7%) | 36 (47.4%) | 43 (51.8%) | 0.629 |
| III/IV | 74 (46.5%) | 37 (48.7%) | 37 (44.6%) |  |
| **Risk factors** |  |  |  |  |
| Diabetes mellitus | 16 (10.1%) | 8 (10.5%) | 8 (9.6%) | 1.000 |
| Heart failure | 4 (2.5%) | 2 (1.3%) | 2 (2.4%) | 1.000 |
| Alcohol abuse | 7 (4.4%) | 2 (1.3%) | 5 (6.0%) | 0.448 |
| Nicotine consumption | 25 (15.7%) | 10 (13.2%) | 15 (18.1%) | 0.515 |
| **Tumor features** |  |  |  |  |
| Synchronous | 132 (83.0%) | 65 (85.5%) | 67 (80.7%) | 0.527 |
| Metachronous | 27 (17.0%) | 11 (14.5%) | 16 (19.3%) |  |
| Bilobar | 115 (72.3%) | 60 (78.9%) | 55 (66.3%) | 0.079 |
| KRAS |  |  |  |  |
| Mutant | 68 (42.8%) | 36 (47.4%) | 32 (38.6%) | 0.612 |
| Wilde-Type | 71 (44.7%) | 34 (44.7%) | 37 (44.6%) |  |
| NA | 20 (12.6%) | 6 (7.9%) | 14 (16.9%) |  |
| T (Category) |  |  |  |  |
| I/II | 20 (12.6%) | 11 (14.5%) | 9 (10.8%) | 0.633 |
| III/IV | 134 (84.3%) | 63 (82.9%) | 71 (85.5%) |  |
| X | 5 (3.1%) | 2 (2.6%) | 3 (3.6%) |  |
| N (Category) |  |  |  |  |
| 0 | 48 (30.2%) | 24 (31.6%) | 24 (28.9%) | 0.729 |
| I/ II | 106 (66.7%) | 49 (64.5%) | 57 (68.7%) |  |
| X | 5 (3.1%) | 3 (3.9%) | 2 (2.4%) |  |
| Perineural invasion | 31 (19.5%) | 19 (25%) | 12 (14.5%) | 0.095 |
| R status |  |  |  |  |
| R0 | 132 (83.0%) | 62 (81.6%) | 70 (84.3%) | 0.659 |
| R1/2 | 23 (14.5%) | 12 (15.8%) | 11 (13.3%) |  |
| X | 4 (2.5%) | 2 (1.3%) | 2 (2.4%) |  |
| Elevated CEA   - before neoadjuvance - before surgery | 39 (24.5%)  69 (43.4%) | 21 (27.6%)  33 (43.4%) | 18 (21.7%)  36 (43.4%) | 0.397  1.000 |
| **Neoadjuvant treatment** |  |  |  |  |
| Indication   - Neoadjuvant - Conversion - unknown | 137 (86.2%)  17 (10.7%)  5 (3.1%) | 61 (80.3%)  12 (15.8%)  3 (3.9%) | 76 (91.6%)  5 (6.0%)  2 (2.4%) | 0.127 |
| Neoadjuvant protocol   - FOLFOX - FOLFIRI - FOLFOXIRI - Panitumumab/Cetuximab - Bevacizumab | 58 (36.5%)  41 (25.8%)  29 (18.2%)  45 (28.3%)  53 (33.3%) | 21 (27.6%)  25 (32.9%)  19 (25%)  20 (26.3%)  30 (39.5%) | 37 (44.6%)  16 (19.3%)  10 (12.0%)  25 (30.1%)  23 (27.7%) | **0.032**  0.069  **0.041**  0.603  0.132 |
| Cycles | 6 (5-8) | 6 (6-8) | 6 (5-8) | 0.628 |
| Premature termination   - Progression - Side effects | 6 (3.8%)  19 (11.9%) | 4 (5.3%)  7 (9.2%) | 2 (2.4%)  12 (14.5%) | 0.427  0.338 |
| Neoadjuvant radiotherapy | 22 (13.8%) | 7 (9.2%) | 15 (18.1%) | 0.113 |
| Time to surgery (days) | 42 (29-60) | 28 (22-34) | 59 (49-79) | <0.001 |
| **Type of liver resection** |  |  |  |  |
| Non-anatomical | 73 (45.9%) |  |  | 0.871 |
| Anatomical   - Hemihepatectomy - Extended hemihepatectomy - Bisegmentectomy - Mesohepatectomy | 43 (27.0%)  26 (16.4%)  16 (10.1%)  1 (0.6%) | 23 (30.3%)  13 (17.1%)  6 (7.9%)  1 (1.3%) | 20 (24.1%)  13 (15.7%)  10 (12.0%)  0 (-) | 0.475  0.833  0.438  0.478 |
| Liver first operation | 11 (6.9%) | 7 (9.2%) | 4 (4.8%) | 0.361 |
| Simultaneous operation | 61 (38.4%) | 28 (36.8%) | 33 (39.8%) | 0.725 |
| Minimally invasive | 26 (16.4%) | 12 (15.8%) | 14 (16.9%) | 1.000 |
| Conventional open surgery | 128 (80.5%) | 61 (80.3%) | 67 (80.7%) |  |
| Two-staged hepatectomy | 29 (18.2%) | 16 (21.1%) | 13 (15.7%) | 0.419 |
| Follow-up time (months) | 31.6 (20.8-51.1) | 27.1 (20.3-45.9) | 35.0 (21.5-55.6) | 0.508 |

**Table 2.** Postoperative outcomes in patients undergoing resection of colorectal liver metastases (CRLM) stratified by TTS ≤ 41 days (n = 76) versus ≥ 42 days (n = 83; total n = 159).

Data are presented as median (interquartile range) for continuous variables and number (percentage) for categorical variables. P-values compare the two TTS groups using the Mann–Whitney U test for continuous outcomes and χ² or Fisher’s exact test for categorical outcomes, as appropriate.

Abbreviations: ICU, intensive care unit; CCI, Comprehensive Complication Index; RFS, recurrence-free survival; OS, overall survival; CRLM, colorectal liver metastases; PHLF, postoperative hepatic liver failure.

|  | **Total**  **[n=159]** | **Shorter TTS**  **[n=76]** | **Longer TTS**  **[n=83]** |  |
| --- | --- | --- | --- | --- |
| **Postoperative Outcomes** | Median (IQR) or Number (Percentage) | | | P |
| Length of hospital stay [d] | 13.5 (9-23) | 15 (10-28) | 13 (9-21) | 0.242 |
| Length of ICU stay [d] | 1.0 (0-3.0) | 1 (0-4.0) | 2 (0-2.5) | 0.073 |
| **Complications** |  |  |  |  |
| Surgical Site Infection | 41 (25.7%) | 24 (31.6%) | 17 (20.5%) | 0.105 |
| Bleeding | 15 (9.4%) | 8 (10.5%) | 7 (8.4%) | 0.609 |
| Biliary leakage | 45 (28.3%) | 23 (30.3%) | 22 (26.5%) | 0.598 |
| PHLF | 6 (3.6%) | 4 (5.3%) | 2 (2.4%) | 0.421 |
| Reoperation | 26 (16.3%) | 12 (15.8%) | 14 (16.9%) | 1.000 |
| Clavien Dindo ≥ 3a | 55 (34.6%) | 29 (38.2%) | 26 (31.3%) | 0.402 |
| CCI | 22.6 (8.7 - 34.8) | 29.2 (8.7 – 41.1) | 22.6 (8.7 - 30.8) | 0.014 |
| CCI ≥ 30 | 63 (40.4%) | 37 (48.7%) | 26 (31.3%) | 0.023 |
| 30 Days Readmission | 26 (16.3%) | 17 (22.4%) | 9 (10.8%) | 0.051 |
| 90 Days Readmission | 24 (15.1%) | 15 (19.7 %) | 9 (10.8%) | 0.120 |
| 90 Days Mortality | 1 (0.6%) | 1 (1.3%) | 0 (-) | 0.478 |
| **Recurrence** |  |  |  |  |
| - Liver | 96 (60.4%) | 51 (67.1%) | 45 (54.2%) | 0.028 |
| - Lung | 26 (16.4%) | 12 (15.8%) | 14 (16.9%) | 1.000 |
| - Peritoneum | 5 (3.1%) | 4 (5.3%) | 3 (3.6%) | 0.710 |
| - Local recurrence | 5 (3.1%) | 2 (2.6%) | 3 (3.6%) | 1.000 |
| Median RFS in months | 9.3 (4.8-25.2) | 8.4 (4.7-19.7) | 9.9 (4.9-35.0) | 0.275 |
| Median overall survival in months | 28.5 (16.6-48.8) | 26.42 (19.0-44.5) | 31.3 (14.4-52.5) | 0.530 |

**Table 3.** Univariable and Multivariable Logistic Regression Analyses for Predictors of Clinically Significant Postoperative Morbidity (CCI ≥30). **(A)** Logistic regression using a dichotomized time-to-surgery (TTS) variable (≥42 days vs. <42 days). **(B)** Logistic regression using TTS as a continuous variable (per day increase). **(C)** Subgroup analysis restricted to patients undergoing major liver resection, using dichotomized TTS (≥42 days vs. <42 days). **(D)** Subgroup analysis restricted to patients undergoing major liver resection, using TTS as a continuous variable (per day increase).

Odds ratios (OR), 95% confidence intervals (CI), and P-values are reported for both univariable and multivariable analyses.

| **(A)** | Univariable | | | Multivariable | | |
| --- | --- | --- | --- | --- | --- | --- |
|  | OR | 95% CI | P | OR | 95% CI | P |
| Time to surgery ≥42d (1) | 0.464 | 0.242 - 0.890 | 0.021 | 0.355 | 0.127-0.992 | 0.048 |
| Age | 0.999 | 0.971-1.027 | 0.929 | 1.020 | 0.971-1.072 | 0.426 |
| ASA III/IV (1) vs. I/II | 0.999 | 0.520 - 1.918 | 0.998 | 0.523 | 0.178-1.537 | 0.239 |
| Female (1) vs. male | 0.633 | 0.318 - 1.260 | 0.193 | 0.470 | 0.161-1.375 | 0.168 |
| BMI | 1.066 | 0.993 - 1.144 | 0.076 | 1.000 | 0.915-1.093 | 0.994 |
| Synchronous (1) vs. metachronous | 3.594 | 1.282 - 10.079 | 0.015 | 0.622 | 0.135-2.876 | 0.544 |
| Bilobar (1) vs. unilobar | 3.054 | 1.377 - 6.775 | 0.006 | 4.576 | 1.155-18.132 | 0.03 |
| Planned termination of chemotherapy (1) vs. premature termination | 1.128 | 0.417 - 3.054 | 0.812 | 0.384 | 0.071-2.071 | 0.266 |
| Chemotherapy cycles | 1.090 | 0.981 - 1.213 | 0.110 | 1.189 | 1.030-1.373 | 0.018 |
| Year of surgery | 1.019 | 0.846 - 1.228 | 0.842 | 1.110 | 0.819-1.504 | 0.500 |
| Minimal invasive (1) vs. open approach | 0.254 | 0.082 - 0.789 | 0.018 | 0.128 | 0.012-1.412 | 0.093 |
| Major (1) vs. minor resection | 3.412 | 1.748 - 6.661 | <0.001 | 5.487 | 1.846-16.307 | 0.002 |
| 2-staged hepatectomy (1) | 2.140 | 0.946 - 4.844 | 0.168 | 0.644 | 0.188-2.206 | 0.484 |
| Bevacizumab (y vs. n) | 0.978 | 0.482-1.982 | 0.950 | 0.451 | 0.148-1.373 | 0.161 |

| **(B)** | Univariable | | | Multivariable | | |
| --- | --- | --- | --- | --- | --- | --- |
|  | OR | 95% CI | P | OR | 95% CI | P |
| Time to surgery | 0.986 | 0.973-1.000 | 0.045 | 0.985 | 0.965-1.005 | 0.136 |
| Age | 0.999 | 0.971-1.027 | 0.929 | 1.025 | 0.976-1.077 | 0.318 |
| ASA III/IV (1) vs. I/II | 0.999 | 0.520 - 1.918 | 0.998 | 0.563 | 0.195-1.624 | 0.288 |
| Female (1) vs. male | 0.633 | 0.318 - 1.260 | 0.193 | 0.435 | 0.151-1.257 | 0.124 |
| BMI | 1.066 | 0.993 - 1.144 | 0.076 | 1.008 | 0.923-1.100 | 0.864 |
| Synchronous (1) vs. metachronous | 3.594 | 1.282 - 10.079 | 0.015 | 0.603 | 0.133-2.736 | 0.512 |
| Bilobar (1) vs. unilobar | 3.054 | 1.377 - 6.775 | 0.006 | 4.606 | 1.186-17.898 | 0.027 |
| Planned termination of chemotherapy (1) vs. premature termination | 1.128 | 0.417 - 3.054 | 0.812 | 0.448 | 0.084-2.374 | 0.345 |
| Chemotherapy cycles | 1.090 | 0.981 - 1.213 | 0.110 | 1.173 | 1.017-1.354 | 0.029 |
| Year of surgery | 1.019 | 0.846 - 1.228 | 0.842 | 1.081 | 0.800-1.461 | 0.613 |
| Minimal invasive (1) vs. open approach | 0.254 | 0.082 - 0.789 | 0.018 | 0.148 | 0.014-1.569 | 0.113 |
| Major (1) vs. minor resection | 3.412 | 1.748 - 6.661 | <0.001 | 5.125 | 1.776-14.788 | 0.003 |
| 2-staged hepatectomy (1) | 2.140 | 0.946 - 4.844 | 0.168 | 0.748 | 0.225-2.487 | 0.635 |
| Bevacizumab (y vs. n) | 0.978 | 0.482-1.982 | 0.950 | 0.463 | 0.153-1.402 | 0.173 |

| **(C)** | Univariable | | | Multivariable | | |
| --- | --- | --- | --- | --- | --- | --- |
|  | OR | 95% CI | P | OR | 95% CI | P |
| Time to surgery ≥42d (1) | 0.190 | 0.068-0.536 | 0.002 | 0.069 | 0.006-0.778 | 0.031 |
| Age | 1.418 | 0.756-2.659 | 0.277 | 0.961 | 0.852-1.084 | 0.516 |
| ASA III/IV (1) vs. I/II | 0.818 | 0.432-1.551 | 0.539 | 0.447 | 0.058-3.465 | 0.441 |
| Female (1) vs. male | 1.248 | 0.649-2.400 | 0.506 | 0.263 | 0.032-2.144 | 0.212 |
| BMI | 0.607 | 0.323-1.141 | 0.121 | 0.959 | 0.772-1.190 | 0.701 |
| Synchronous (1) vs. metachronous | 6.727 | 1.309-34.572 | 0.022 | 0.174 | 0.006-5.153 | 0.312 |
| Bilobar (1) vs. unilobar | 2.000 | 0.610-6.561 | 0.253 | 1.569 | 0.132-18.681 | 0.722 |
| Planned termination of chemotherapy (1) vs. premature termination | 1.128 | 0.417 - 3.054 | 0.812 | 51.025 | 0.518-5030.222 | 0.093 |
| Chemotherapy cycles | 1.180 | 0.947-1.469 | 0.140 | 1.34 | 0.746-2.407 | 0.328 |
| Year of surgery |  |  |  | 3.794 | 1.202-11.978 | 0.023 |
| Minimal invasive (1) vs. open approach | 0.254 | 0.082 - 0.789 | 0.018 | 0.015 | 0.000-1.219 | 0.061 |
| 2-staged hepatectomy (1) | 2.054 | 0.709-5.951 | 0.185 | 1.698 | 0.222-12.953 | 0.610 |
| Bevacizumab (y vs. n) | 1.037 | 0.368-2.921 | 0.945 | 0.419 | 0.041-4.331 | 0.465 |

| **(D)** | Univariable | | | Multivariable | | |
| --- | --- | --- | --- | --- | --- | --- |
|  | OR | 95% CI | P | OR | 95% CI | P |
| Time to surgery | 0.958 | 0.931-0.986 | 0.003 | 0.953 | 0.908-1.001 | 0.057 |
| Age | 0.999 | 0.971-1.027 | 0.929 | 0.981 | 0.8801.094 | 0.730 |
| ASA III/IV (1) vs. I/II | 0.999 | 0.520 - 1.918 | 0.998 | 0.545 | 0.078-3.829 | 0.542 |
| Female (1) vs. male | 0.633 | 0.318 - 1.260 | 0.193 | 0.373 | 0.055-2.530 | 0.313 |
| BMI | 1.066 | 0.993 - 1.144 | 0.076 | 0.993 | 0.818-1.205 | 0.942 |
| Synchronous (1) vs. metachronous | 3.594 | 1.282 - 10.079 | 0.015 | 0.285 | 0.013-6.127 | 0.423 |
| Bilobar (1) vs. unilobar | 3.054 | 1.377 - 6.775 | 0.006 | 2.354 | 0.201-27.621 | 0.496 |
| Planned termination of chemotherapy (1) vs. premature termination | 1.128 | 0.417 - 3.054 | 0.812 | 24.278 | 0.498-1184.321 | 0.108 |
| Chemotherapy cycles | 1.090 | 0.981 - 1.213 | 0.110 | 1.438 | 0.809-2.555 | 0.216 |
| Year of surgery | 1.019 | 0.846 - 1.228 | 0.842 | 2.809 | 1.1406.921 | 0.025 |
| Minimal invasive (1) vs. open approach | 0.254 | 0.082 - 0.789 | 0.018 | 0.053 | 0.002-1.838 | 0.105 |
| 2-staged hepatectomy (1) | 2.140 | 0.946 - 4.844 | 0.168 | 2.308 | 0.340-15.679 | 0.392 |
| Bevacizumab (y vs. n) | 1.037 | 0.368-2.921 | 0.945 | 0.494 | 0.056-4.384 | 0.526 |

**Table 4.** Multivariable Cox Regression Analyses for Liver-Specific Recurrence-Free Survival (RFS). **(A)** Analysis using TTS as a dichotomized variable (≥42 days vs. <42 days). **(B)** Analysis using TTS as a continuous variable (scaled per day). Hazard ratios (HR), 95% confidence intervals (CI), and P-values are reported. *CEA - Carcinoembryonic antigen; HR – Hazard ratio.

| **(A)** | **Univariable** | | | **Multivariable** | | |
| --- | --- | --- | --- | --- | --- | --- |
|  | HR | 95% CI | P | HR | 95% CI | P |
| **Time to surgery ≥42d (1)** | 0.688 | 0.460-1.030 | 0.069 | 0.888 | 0.492-1.603 | 0.694 |
| Age | 0.993 | 0.977-1.010 | 0.415 | 0.996 | 0.973-1.020 | 0.768 |
| Female (1) vs. male | 0.685 | 0.440-1.066 | 0.093 | 0.602 | 0.326-1.113 | 0.106 |
| Synchronous (1) vs. metachronous | 0.881 | 0.507-1.530 | 0.653 | 1.620 | 0.755-3.476 | 0.216 |
| Bilobar (1) vs. unilobar | 1.487 | 0.921-2.402 | 0.105 | 0.863 | 0.416-1.790 | 0.962 |
| Preoperative CEA levels | 1.829 | 1.190-2.813 | 0.006 | 1.004 | 0.997-1.010 | 0.275 |
| BRAF mutation (1) | 0.757 | 0.280-2.044 | 0.583 | 1.020 | 0.122-8.502 | 0.986 |
| Planned termination of chemotherapy (1) vs. premature termination | 1.343 | 0.727-2.482 | 0.347 | 0.709 | 0.289-1.740 | 0.453 |
| Chemotherapy cycles | 0.937 | 0.875-1.003 | 0.062 | 1.015 | 0.920-1.120 | 0.764 |
| 2-staged hepatectomy (1) | 1.669 | 1.023-2.722 | 0.040 | 2.228 | 1.146-4.329 | 0.018 |
| R-stage ≥1 | 1.760 | 1.037-2.987 | 0.036 | 1.377 | 0.594-3.192 | 0.456 |
| FOLFIRI (1) | 0.371 | 0.128-1.074 | 0.068 | 0.617 | 0.335-1.137 | 0.121 |
| Panitumumab/Cetuximab (1) | 1.723 | 1.045-2.840 | 0.033 | 1.732 | 0.902-3.326 | 0.098 |
| **(B)** | **Univariable** | | | **Multivariable** | | |
|  | HR | 95% CI | P | HR | 95% CI | P |
| **Time to surgery [days]** | 1.006 | 0.998-1.015 | 0.156 | 0.999 | 0.985-1.013 | 0.867 |
| Age | 0.993 | 0.977-1.010 | 0.415 | 0.997 | 0.974-1.021 | 0.816 |
| Female (1) vs. male | 0.685 | 0.440-1.066 | 0.093 | 0.590 | 0.322-1.081 | 0.088 |
| Synchronous (1) vs. metachronous | 0.881 | 0.507-1.530 | 0.653 | 1.618 | 0.753-3.475 | 0.218 |
| Bilobar (1) vs. unilobar | 1.487 | 0.921-2.402 | 0.105 | 0.871 | 0.419-1.812 | 0.712 |
| Preoperative CEA levels | 1.829 | 1.190-2.813 | 0.006 | 1.004 | 0.997-1.010 | 0.285 |
| BRAF mutation | 0.757 | 0.280-2.044 | 0.583 | 1.059 | 0.128-8.747 | 0.958 |
| Planned termination of chemotherapy (1) vs. premature termination | 1.343 | 0.727-2.482 | 0.347 | 0.730 | 0.300-1.777 | 0.488 |
| Chemotherapy cycles | 0.937 | 0.875-1.003 | 0.062 | 1.016 | 0.921-1.121 | 0.750 |
| 2-staged hepatectomy (1) | 1.669 | 1.023-2.722 | 0.040 | 2.282 | 1.188-4.386 | 0.013 |
| R-stage ≥1 | 1.760 | 1.037-2.987 | 0.036 | 1.430 | 0.632-3.235 | 0.391 |
| FOLFIRI (1) | 0.371 | 0.128-1.074 | 0.068 | 0.613 | 0.332-1.134 | 0.119 |
| Panitumumab/Cetuximab (1) | 1.723 | 1.045-2.840 | 0.033 | 1.753 | 0.904-3.401 | 0.097 |
