# Supplementary material for: Delaying surgery beyond six weeks after systemic therapy reduces postoperative morbidity without evidence of impaired oncologic outcomes in colorectal liver metastases
Source: BMC Cancer. 2026 Jun 30;26:786. doi: 10.1186/s12885-026-16386-4 (PMC13317304; doi:10.1186/s12885-026-16386-4)
Supplement: Supplementary file 4 — Supplementary Material 4. [file 12885_2026_16386_MOESM4_ESM.docx]

**Supplementary Figure 1. Distribution of Time‑to‑Surgery Following Neoadjuvant Chemotherapy.** Histogram showing the frequency of exact intervals (in days) between completion of neoadjuvant chemotherapy and hepatic resection in 159 patients.

**Supplementary Figure 2. Effects of Time‑to‑Surgery and Resection Type on Postoperative Morbidity.** Estimated marginal means of log₁₀(CCI + 1) with ± 95 % CI for minor (blue) and major (red) resections, stratified by TTS group (< 42 days vs. ≥ 42 days). Lines connect the two TTS categories within each resection type.

**Supplementary Table 1**

Multivariable logistic regression predicting clinically relevant postoperative morbidity (Comprehensive Complication Index > 30) including restricted cubic spline terms for Time‑to‑Surgery (knots at 12, 42 and 89 days) and adjustment for key clinical covariates. The linear term (x) and two spline components (SPL2, SPL3) model the continuous effect of TTS in days. Other covariates include bilobar metastases, major resection, age at time of surgery, synchronous versus metachronous presentation, body mass index, FOLFOXIRI regimen, regular treatment completion, and ASA class. Exp(B) denotes odds ratios with 95 % confidence intervals.

|  | Multivariabel | | |
| --- | --- | --- | --- |
|  | OR | 95% CI | P |
| x | 0.995 | 0.940 - 1.053 | 0.857 |
| SPL2 | 1.000 | 1.000 - 1.000 | 0.543 |
| SPL3 | 1.000 | 1.000 - 1.000 | 0.429 |
| Bilobar (1) vs. unilobar | 2.429 | 0.958 - 6.157 | 0.061 |
| Major resection (1) vs. Minor resection | 2.929 | 1.370 - 6.263 | 0.006 |
| Age | 1.025 | 0.987 - 1.064 | 0.203 |
| Synchronous (1) vs. metachronous | 0.206 | 0.057 - 0.738 | 0.015 |
| BMI | 1.037 | 0.961 - 1.120 | 0.345 |
| FOLFOXIRI (1) vs. other regimens | 1.170 | 0.457 - 2.996 | 0.743 |
| Planned termination of chemotherapy vs. termination due to side effects or progress | 1.034 | 0.342 - 3.132 | 0.952 |
| ASA III/IV (1) vs. I/II | 0.980 | 0.454 - 2.117 | 0.960 |

**Supplementary Table 2.** Adjusted analyses of the effect of time-to-surgery (TTS) and type of resection on postoperative morbidity (log₁₀(CCI + 1)).

| **A.** **Adjusted marginal means** of log₁₀(CCI + 1) (± standard error [SE], 95% confidence interval [CI]) according to TTS (< 42 vs. ≥ 42 days) and extent of resection. | | | | | | | |
| --- | --- | --- | --- | --- | --- | --- | --- |
| **Type of resection** | | **TTS group** | | **Mean logCCI ± Std. Error** | | | **95% CI** |
| Minor | | <42 days | | 1.28 (±0.05) | | | 1.18 – 1.38 |
| Minor | | ≥42 days | | 1.26 (±0.05) | | | 1.16 – 1.36 |
| Major | | <42 days | | 1.55 (±0.06) | | | 1.43 – 1.67 |
| Major | | ≥42 days | | 1.38 (±0.06) | | | 1.26 – 1.50 |
| **B.** **Multivariable linear regression coefficients** (B, 95% CI, p-value) for delayed surgery (TTS ≥ 42 days), major resection (at TTS < 42 days), and their interaction, derived from the IPTW-weighted model. | | | | | | | |
|  | | | **B** | | **95% CI** | | **p-Value** |
| TTS ≥ 42 days (Minor resection) | | | –0.014 | | –0.123 to 0.095 | | 0.798 |
| Major resection (at TTS < 42 d) | | | +0.304 | | +0.187 to 0.421 | | <0.001 |
| TTS × Major resection interaction | | | –0.181 | | –0.344 to –0.018 | | 0.030 |
| **C. Simple-slope contrasts** comparing delayed (≥ 42 days) versus early (< 42 days) surgery within each resection subgroup, with back-transformed CCI means. | | | | | | | |
| **Type of resection** | **TTS group** | | **Mean Difference** | | | **p-Value** | **Mean CCI*** |
| Minor | <42 days vs. ≥42 days | | -0.02 | | | 0.80 | 18 vs. 19 |
| Major | <42 days vs. ≥42 days | | -0.17 | | | 0.03 | 24 vs. 35 |
| *Back-transformed means calculated as 10^(Mean logCCI) – 1. | | | | | | | |

**Supplementary Table 3**

**Univariable and Multivariable Logistic Regression Analyses of Predictors for Overall Survival.** This table summarizes the results of univariable and multivariable logistic regression analyses assessing potential predictors of overall survival. Odds ratios (OR), 95% confidence intervals (CI), and P-values are reported. Variables with P < 0.10 or deemed clinically relevant were considered for inclusion in multivariable modeling.

|  | **Univariable** | | | **Multivariable** | | |
| --- | --- | --- | --- | --- | --- | --- |
|  | **HR** | **95% CI** | **P** | **HR** | **95% CI** | **P** |
| **Demographic data** |  |  |  |  |  |  |
| Age | 1.025 | 1.005-1.046 | **0.014** | 1.048 | 1.012-1.085 | **0.009** |
| Gender (male vs. female) | 1.162 | 0.752-1.795 | 0.498 |  |  |  |
| BMI | 0.977 | 0.933-1.022 | 0.308 |  |  |  |
| ASA (III/IV vs. I/II) | 1.004 | 0.654-1.541 | 0.987 |  |  |  |
| **Preoperative labaratory data** |  |  |  |  |  |  |
| alkaline phosphatase | 1.003 | 1.001-1.005 | **0.005** | 1.001 | 0.992-1.010 | 0.802 |
| INR | 1000 | 1.000-1.000 | 0.206 |  |  |  |
| ALT | 0.998 | 0.990-1.006 | 0.63 |  |  |  |
| AST | 1.004 | 0.994-1.015 | 0.413 |  |  |  |
| GGT | 1.002 | 1.000-1.003 | **0.007** | 1.002 | 0.997-1.006 | 0.496 |
| CRP | 1.07 | 0.686-1.670 | 0.765 |  |  |  |
| Total Bilirubin | 1.621 | 0.809-3.248 | 0.173 |  |  |  |
| Albumin | 1.013 | 0.947-1.083 | 0.71 |  |  |  |
| **Tumor markers** |  |  |  |  |  |  |
| Preoperative CEA (Elevated vs. Normal) | 2.333 | 1.477-3.684 | **<0.001** | 1.133 | 0.463-2.773 | 0.784 |
| Preneoadjuvant CEA (Elevated vs. Normal) | 2.125 | 0.955-4.726 | **0.065** | 1.602 | 0.588-4.370 | 0.357 |
| **Tumor Features** |  |  |  |  |  |  |
| Synchronous vs. Metachronous | 1.528 | 0.917-2.547 | 0.104 |  |  |  |
| Bilobar (Yes vs. No) | 1.361 | 0.839-2.209 | 0.212 |  |  |  |
| KRAS (Mutant vs. Wild-Typ) | 0.71 | 0.361-1.396 | 0.321 |  |  |  |
| T > II | 1.83 | 0.844-3.970 | 0.126 |  |  |  |
| N (+ vs. -) | 2.328 | 1.331-4.072 | **0.003** | 1.851 | 0.724-4.732 | 0.198 |
| Perineural invasion (+ vs. -) | 0.831 | 0.349-1.977 | 0.675 |  |  |  |
| R Status (+ vs. -) | 0.841 | 0.550-1.285 | 0.423 |  |  |  |
| **Neoadjuvant treatment** |  |  |  |  |  |  |
| Neoadjuvant radiotherapy (1) | 1.45 | 0.841-2.500 | 0.182 |  |  |  |
| Time to surgery ≥42d (1) | 0.916 | 0.601-1.395 | 0.682 |  |  |  |
| **Type of liver resection** |  |  |  |  |  |  |
| Non-anatomical vs. Anatomical | 0.822 | 0.539-1.255 | 0.365 |  |  |  |
| Minimally invasive vs. open | 1.068 | 0.262-4.364 | 0.927 |  |  |  |
| Two-Staged Hepatectomy (1) | 1.423 | 0.836-2.422 | 0.194 |  |  |  |
| Liver-first if Synchronous (1) | 1.713 | 0.776-3.780 | 0.183 |  |  |  |

**Supplementary Table 4.** Sensitivity analyses of alternative TTS cut-offs for prediction of clinically significant postoperative morbidity (CCI ≥30).

Univariable and multivariable logistic regression models evaluating TTS dichotomized at 35 days (A), 49 days (B), and 56 days (C). Odds ratios (OR), 95% confidence intervals (CI), and P-values are shown. Multivariable models were adjusted for demographic, tumor-related, surgical, and treatment-related covariates including bevacizumab exposure.

| **(A)** | Univariable | | | Multivariable | | |
| --- | --- | --- | --- | --- | --- | --- |
|  | OR | 95% CI | P | OR | 95% CI | P |
| Time to surgery ≥35d (1) | 0.595 | 0.-308-1-152 | 0.124 | 0.493 | 0.181-1.342 | 0.166 |
| Age | 0.999 | 0.971-1.027 | 0.929 | 1.021 | 0.973-1.072 | 0.397 |
| ASA III/IV (1) vs. I/II | 0.999 | 0.520 - 1.918 | 0.998 | 0.579 | 0.205-1.639 | 0.304 |
| Female (1) vs. male | 0.633 | 0.318 - 1.260 | 0.193 | 0.459 | 0.159-1.325 | 0.15 |
| BMI | 1.066 | 0.993 - 1.144 | 0.076 | 1.012 | 0.9271.104 | 0.798 |
| Synchronous (1) vs. metachronous | 3.594 | 1.282 - 10.079 | 0.015 | 0.547 | 0.121-2.476 | 0.434 |
| Bilobar (1) vs. unilobar | 3.054 | 1.377 - 6.775 | 0.006 | 4.586 | 1.174-17.917 | 0.028 |
| Planned termination of chemotherapy (1) vs. premature termination | 1.128 | 0.417 - 3.054 | 0.812 | 0.490 | 0.099-2.437 | 0.384 |
| Chemotherapy cycles | 1.090 | 0.981 - 1.213 | 0.110 | 1.177 | 1.023-1.356 | 0.023 |
| Year of surgery | 1.019 | 0.846 - 1.228 | 0.842 | 1.098 | 0.813-1.484 | 0.541 |
| Minimal invasive (1) vs. open approach | 0.254 | 0.082 - 0.789 | 0.018 | 0.179 | 0.018-1.813 | 0.145 |
| Major (1) vs. minor resection | 3.412 | 1.748 - 6.661 | <0.001 | 5.345 | 1.836-15.560 | 0.002 |
| 2-staged hepatectomy (1) | 2.140 | 0.946 - 4.844 | 0.168 | 0.760 | 0.228-2.535 | 0.655 |
| Bevacizumab (y vs. n) | 0.978 | 0.482-1.982 | 0.950 | 0.440 | 0.145-1.339 | 0.148 |

| **(B)** | Univariable | | | Multivariable | | |
| --- | --- | --- | --- | --- | --- | --- |
|  | OR | 95% CI | P | OR | 95% CI | P |
| Time to surgery ≥49d (1) | 0.518 | 0.265-1.011 | 0.054 | 0.346 | 0.122-0.985 | 0.047 |
| Age | 0.999 | 0.971-1.027 | 0.929 | 1.027 | 0.978-1.097 | 0.283 |
| ASA III/IV (1) vs. I/II | 0.999 | 0.520 - 1.918 | 0.998 | 0.517 | 0.175-1.530 | 0.233 |
| Female (1) vs. male | 0.633 | 0.318 - 1.260 | 0.193 | 0.437 | 0.148-1.285 | 0.132 |
| BMI | 1.066 | 0.993 - 1.144 | 0.076 | 1.004 | 0.919-1.096 | 0.931 |
| Synchronous (1) vs. metachronous | 3.594 | 1.282 - 10.079 | 0.015 | 0.605 | 0.130-1.2.813 | 0.522 |
| Bilobar (1) vs. unilobar | 3.054 | 1.377 - 6.775 | 0.006 | 4.765 | 1.186-19.141 | 0.028 |
| Planned termination of chemotherapy (1) vs. premature termination | 1.128 | 0.417 - 3.054 | 0.812 | 0.433 | 0.079-2.361 | 0.333 |
| Chemotherapy cycles | 1.090 | 0.981 - 1.213 | 0.110 | 1.174 | 1.013-1.360 | 0.033 |
| Year of surgery | 1.019 | 0.846 - 1.228 | 0.842 | 1.121 | 0.826-1.522 | 0.463 |
| Minimal invasive (1) vs. open approach | 0.254 | 0.082 - 0.789 | 0.018 | 0.107 | 0.009-1.230 | 0.073 |
| Major (1) vs. minor resection | 3.412 | 1.748 - 6.661 | <0.001 | 5.663 | 1.903-16.848 | 0.002 |
| 2-staged hepatectomy (1) | 2.140 | 0.946 - 4.844 | 0.168 | 0.692 | 0.204-2.353 | 0.556 |
| Bevacizumab (y vs. n) | 0.978 | 0.482-1.982 | 0.950 | 0.476 | 0.155-1.459 | 0.194 |

| **(C)** | Univariable | | | Multivariable | | |
| --- | --- | --- | --- | --- | --- | --- |
|  | OR | 95% CI | P | OR | 95% CI | P |
| Time to surgery ≥56d (1) | 0.412 | 0.197-0.862 | 0.019 | 0.349 | 0.116-1.050 | 0.061 |
| Age | 0.999 | 0.971-1.027 | 0.929 | 1.023 | 0.975-1.074 | 0.353 |
| ASA III/IV (1) vs. I/II | 0.999 | 0.520 - 1.918 | 0.998 | 0.542 | 0.185-1.587 | 0.264 |
| Female (1) vs. male | 0.633 | 0.318 - 1.260 | 0.193 | 0.449 | 0.155-1.307 | 0.142 |
| BMI | 1.066 | 0.993 - 1.144 | 0.076 | 1.000 | 0.915-1.093 | 0.994 |
| Synchronous (1) vs. metachronous | 3.594 | 1.282 - 10.079 | 0.015 | 0.705 | 0.151-3.293 | 0.657 |
| Bilobar (1) vs. unilobar | 3.054 | 1.377 - 6.775 | 0.006 | 4.579 | 1.167-17.973 | 0.029 |
| Planned termination of chemotherapy (1) vs. premature termination | 1.128 | 0.417 - 3.054 | 0.812 | 0.492 | 0.096-2.535 | 0.397 |
| Chemotherapy cycles | 1.090 | 0.981 - 1.213 | 0.110 | 1.175 | 1.014-1.361 | 0.032 |
| Year of surgery | 1.019 | 0.846 - 1.228 | 0.842 | 1.083 | 0.799-1.467 | 0.607 |
| Minimal invasive (1) vs. open approach | 0.254 | 0.082 - 0.789 | 0.018 | 0.119 | 0.011-1.337 | 0.085 |
| Major (1) vs. minor resection | 3.412 | 1.748 - 6.661 | <0.001 | 5.249 | 1.801-15.297 | 0.002 |
| 2-staged hepatectomy (1) | 2.140 | 0.946 - 4.844 | 0.168 | 0.768 | 0.230-2.567 | 0.668 |
| Bevacizumab (y vs. n) | 0.978 | 0.482-1.982 | 0.950 | 0.469 | 0.153-1.439 | 0.185 |
